# Biochar Derived from Urban Green Waste Can Enhance the Removal of Cd from Water and Reduce Soil Cd Bioavailability

**DOI:** 10.3390/toxics12010008

**Published:** 2023-12-21

**Authors:** Xiang Li, Paramsothy Jeyakumar, Nanthi Bolan, Lianxi Huang, Muhammad Saqib Rashid, Zhongzhen Liu, Lan Wei, Hailong Wang

**Affiliations:** 1 Key Laboratory of Plant Nutrition, and Fertilizer in South Region, Ministry of Agriculture, Guangdong Key Laboratory of Nutrient Cycling and Farmland Conservation, Institute of Agricultural Resources and Environment, Guangdong Academy of Agricultural Sciences, Guangzhou 510640, China; lixiang142213@163.com (X.L.); hlx4@163.com (L.H.); saqibssr@mail.ustc.edu.cn (M.S.R.); 2 Environmental Sciences, School of Agriculture & Environment, Massey University, Private Bag 11 222, Palmerston North 4442, New Zealand; p.jeyakumar@massey.ac.nz; 3 UWA School of Agriculture and Environment, The University of Western Australia, Perth, WA 6009, Australia; nanthi.bolan@uwa.edu.au; 4 The UWA Institute of Agriculture, The University of Western Australia, Perth, WA 6009, Australia; 5 School of Environmental and Chemical Engineering, Foshan University, Foshan 528000, China; hailong.wang@fosu.edu.cn; 6 Guangdong Provincial Key Laboratory of Integrated Agro-Environmental Pollution Control and Management, Institute of Eco-Environmental and Soil Sciences, Guangdong Academy of Sciences, Guangzhou 510650, China

**Keywords:** urban green waste, adsorption, biochar immobilization, Cd, soil remediation

## Abstract

The beneficial utilization of potentially increasing urban green waste (UGW) is critical for sustainable urban development in China. In this study, UGW was pyrolyzed at different temperatures, and the resulting biochar was used to amend Cd-contaminated soils to grow cabbage. Our results showed that the Cd adsorption capacity of UGW-biochar was positively correlated with the surface area, O/C, and (O+N)/C value of biochar. Furthermore, UGW-biochar was incorporated into three Cd-contaminated soils, including one acidic soil and two neutral soils, to assess its impact on the availability of Cd. The most substantial reduction in the concentration of available Cd was observed in the acidic soil, of the three tested soils. In the neutral soils, a more substantial reduction was found in the heavily Cd-contaminated soil compared to the lightly Cd-contaminated soil. UGW-biochar amendments to the three Cd-contaminated soils resulted in an increase in the cabbage biomass in acidic soil, whereas in neutral soils, it increased in lightly contaminated soils but decreased in heavily contaminated soils. Additionally, the Cd bioaccumulation factor (BCF), translocation factor (TF), and removal efficiency (RE), as impacted by the biochar application, were calculated in the lightly Cd-contaminated soil–cabbage system. The BCF decreased from 5.84 to 3.80 as the dosage of the UGW-biochar increased from 0% to 3%, indicating that the UGW-biochar immobilized Cd and reduced its bioaccumulation in cabbage roots. Based on our investigations, UGW-biochar effectively immobilizes Cd by reducing its mobility and bioavailability in a lightly contaminated environment matrix.

## 1. Introduction

Soil cadmium (Cd) contamination due to mining activities, industrial waste, sewage sludge, and excessive chemical fertilizers and pesticides is a serious challenge worldwide [1]. According to the data from a national pollution census in China, more than 20 million hectares are contaminated by heavy metals [2], and Cd is the most serious contaminant in terms of exceeding the Ministry of Environmental Protection (MEP) limit [3,4]. The main challenge of high Cd accumulation in soil is characterized by its persistent toxicity, non-degradability, and solubility [5,6]. Therefore, high Cd accumulation in agricultural soils poses a threat to human health when ingested through dietary intake. Long-term exposure to Cd can result in a range of health issues, such as kidney dysfunction, osteoporosis, and cancer [7]. Moreover, Cd can disrupt the endocrine system, impair neurological function, and negatively affect reproductive health [8]. Given these serious consequences, addressing Cd contamination in agricultural soils is vital to ensure both environmental sustainability and public health.

Recently, pyrolysis has received increasing attention as one of the environmentally friendly practices that can be used to manage organic waste. Pyrolysis converts organic waste, at limited oxygen conditions, into high-value products, and biochar is one of the leading products of pyrolysis. Biochar is a carbonaceous solid product resulting from slow pyrolysis. Biochar is characterized by a large surface area, high cation exchange capacity, high stability, and a large number of functional groups [9,10]. Several studies have demonstrated that biochar treatment can significantly improve soil’s physical, chemical, and even biological properties, thus promoting plant growth [11,12]. The potential of biochar to reduce Cd toxicity in agricultural soils has been reported in different studies [13,14]. Biochar can directly fix metals in contaminated soils due to its large surface area, rich binding sites, and numerous functional groups [15]. Meanwhile, biochar can improve soil quality and change the microhabitat of soil microbes [16]. The characteristics of biochar, such as its large specific surface area, microporous structure, and rich oxygenation groups, have been attributed to its value in mitigating the bioavailability and eco-toxicological impacts of heavy metals [9,17,18]. While biochar has demonstrated promising results, its effectiveness depends on factors including feedstock, pyrolysis temperature, retention, and atmospheric conditions [19,20]. Several studies have reported varying results from the usage of different organic wastes (rice husk, maize straws, and animal manure) as feedstock materials for the synthesis of biochar [21,22,23].

In recent decades, urban greening waste (UGW), consisting of garden waste, has drastically increased in most cities in China due to rapid urbanization and efficient waste segregation and collection [24]. Shi et al. [25] estimated that the annual production of UGW is more than 14.4 million tons in the provinces of Guangdong, Jiangsu, Shandong, and Zhejiang. The traditional practice of urban greening waste management is through incineration or landfill; however, these methods have proved to significantly contribute to water (ground and surface) and air pollution. Further, this conventional method needs a large area that could potentially be used for other practices, such as farming. Thus, the current practices of UGW management need to be improved to reduce the loss of land through landfills. It is therefore urgent to develop innovative techniques for managing the vast volume of UGW in most Chinese cities [26,27]. UGW is composed of various plant debris, such as trunks, branches, leaves, and grass. Generally, it consists of about 40% cellulose, 20–30% hemicelluloses, and 25–30% lignin [25], although this varies widely depending on the climatic zone and land management practices [28]. There is limited knowledge about the characteristics of the biochar derived from UGW or whether that biochar can play a significant role in remediating heavy metal-contaminated soil.

This study aims to explore the effect of UGW biochar on Cd-contaminated soil for growing cabbage. Specifically, we aim (i) to characterize the biochar prepared with UGW, (ii) to investigate the impacts of the UGW biochar treatment on Cd bioavailability, and (iii) to quantify the remediated plant growth performance using cabbage as a test crop.

## 2. Materials and Methods

### 2.1. Soils and Biochars

This experiment was conducted using three soil types: Udept, Ustalf, and Udult, according to the US Soil Taxonomy, each with different Cd contents. Udept soil was collected from Yunfu (111°03′–112°31′ E, 22°22′–23°19′ N), Ustalf soil from Jiyuan (112°25′–112°33′ E, 35°25′–35°33′ N), and Udult soil from Shaoguan (113°30′–114°02′ E, 24°56′–25°27′ N). The three soils were collected from agricultural land (Yunfu and Jiyuan) or a mining area (Shaoguan) at a 0–20 cm depth, air-dried, and then sieved through a 2 mm mesh. Sub-samples of each soil were analyzed for basic soil properties (Table 1). The pH of biochar and soil were investigated using a 1:5 and 1:10 soil-to-water ratio, respectively, with a pH electrode (In Lab Expert Pro, order No 51343101, Shanghai, China).

The UGW biochar was prepared from bulk portions of the air-dried urban greening waste (shredded to be <2 cm) supplied by the Guangdong Shunde Dadi Landscape and Environmental Engineering Co. in Foshan City, Guangdong Province, China. The pyrolysis temperatures were 350, 450, 550, and 650 °C, and the biomass was pyrolyzed at these temperatures for 3 h. The resulting biochars from the different pyrolysis temperatures were named UGW 350, UGW 450, UGW 550, and UGW 650, respectively. Each biochar type was crushed and passed through a <75 mm mesh to determine its structural elements, porous structure, and surface characteristics. The biochar’s structural elements were quantified using the Elementar of a Vario EL cube. Both the Brunauer–Emmett–Teller surface area (BET) and the total pore volume were calculated from the N_2_ sorption–desorption isotherms using a gas analyzer (Quantachrome Quadrasorb, Boynton Beach, FL, USA). A field emission scanning electron microscopy (SEM) instrument (S-3400N-II, Hitachi, Japan) was used to observe the surface morphology. The functional groups were delineated by Fourier transform infrared spectroscopy (FTIR) (VETREX 70, Bruker, Billerica, MA, USA). The zeta potential (ZP) was determined using a micro-electrophoresis apparatus (JS94G+, Shanghai, China).

### 2.2. Isothermal Adsorption

This experiment was conducted in triplicates using 25 mL vials. Briefly, the batch experiment was conducted by mixing each biochar into various Cd concentrations ranging from 0.06 mg/L to 19.00 mg/L. The mass of UGW350, UGW450, UGW550, and UGW650 were 0.03 g, 0.02 g, 0.015 g, and 0.015 g, respectively. The volume of the solution was 20 mL. The initial pH of the suspension was adjusted to 7.0 by using either 0.01 mol/L HNO_3_ or 0.01 mol/L NaOH. The vials were placed into a constant temperature oscillator and shaken at 150 rpm for 48 h. Then, the suspension was immediately filtered through 0.45 μm pore sized nylon membrane filters (Jinteng Experiment Equipment Co., Ltd., Tianjin, China). Freundlich and Langmuir models were used to describe the Cd adsorption isotherms for biochars. The biochar with the highest affinity to Cd was chosen to be further used in the pot experiments to test its influence on the bioavailability of soil Cd. The adsorbed Cd quantity was calculated with the following Equation (1), outlined by [29]:

(1) $$q=\frac{{\mathrm{Cd}}_{0}-{\mathrm{Cd}}_{e}}{m}\times V$$

where q (mg/g) is the adsorption capacity, ${\mathrm{Cd}}_{0}$ (mg/L) and ${\mathrm{Cd}}_{e}$ (mg/L) are the Cd concentration at the initial and equilibrium test, respectively, V (L) is the volume of the solution, and m (g) is the biochar rate.

### 2.3. Pot Experiment

This pot experiment was conducted in a glasshouse. Before the experiment commenced, three typical soils were collected from Yunfu, Jiyuan, and Shaoguan. The soils were air-dried for 1 week and passed through a 2 mm sieve. Then, each soil type was mixed with two biochar levels, i.e., 0% and 3% (*w*/*w*) of the UGW450. The pots were filled with 3.5 kg of treated or untreated soil. The pot’s size was 25 cm at its top diameter and 20 cm in height. This resulted in a total of 24 pots (3 soils × 2 biochar levels × 4 replications). The soil mixtures then received urea at 100 mg N/kg soil, superphosphate at 80 mg P/kg soil, and K_2_SO_4_ at 100 mg K/kg soil. The soil was watered to 70% water holding capacity (WHC) using distilled water. The soil in the pots was incubated for 2 weeks in a closed chamber (25 °C) to mix the applied treatments with the soil matrix. During the incubation period, all pots were maintained at 70% WHC by weighing them constantly and adding the required amount of distilled water. After 2 weeks of soil incubation, each pot was sown with 10 seeds of Chinese cabbage (*Brassica chinensis* L.). Then, all pots were transferred to the glasshouse and arranged in a randomized complete block design. Chinese cabbage seedlings were thinned to one plant per pot after 8 days of emergence. The average night and day temperatures for the glasshouse were 20 °C and 28 °C, respectively, throughout the 48 days of the cabbage growing period. The Chinese cabbage plants from each pot were harvested after 48 days by separating the root, stem, petiole, and blade samples. The Chinese cabbage samples were measured for their dry matter weight and Cd concentration. The soil samples were also collected and stored at room temperature before assaying the available Cd content, pH, total P, and total N. The available Cd concentration in soils was extracted using diethylenetriaminepentaacetic acid/triethanolamine (DTPA/TEA), and the Cd concentration in the extracts and water was determined with a flame-polarized Zeeman atomic absorption spectrophotometer (Varian AA240FS) [30]. The total Cd concentrations in the soils and cabbage tissues were determined with a flame-polarized Zeeman atomic adsorption spectrophotometer (Varian AA240FS) after H_2_SO_4_/HClO_4_ (2:1 ratio *v*/*v*) digestion [31].

### 2.4. Data Processing and Statistical Analysis

The bioaccumulation factor (BCF), translocation factor (TF), and removal efficiency (RE) of Cd among the plant organs were calculated as follows [32]:

(2) $$\mathrm{BCF}=\frac{C_{\mathrm{root}}}{C_{\mathrm{soil}}}$$

(3) $$\mathrm{TF}=\frac{C_{\mathrm{shoot}}}{C_{\mathrm{root}}}$$

(4) $$\mathrm{RE}=\frac{C_{\mathrm{shoot}}\times M_{\mathrm{shoot}}+C_{\mathrm{root}}\times M_{\mathrm{root}}}{C_{\mathrm{soil}}\times M_{\mathrm{soil}}}$$

where $C_{\mathrm{root}}$, $C_{\mathrm{shoot}}$, and $C_{\mathrm{soil}}$ were the Cd content (mg/g) in the cabbage root, shoot, and soil, respectively, while $M_{\mathrm{shoot}}$, $M_{\mathrm{root}}$, and $M_{\mathrm{soil}}$ were the dry matter of the cabbage root, shoot, and soil, respectively.

All the results of the pot experiments were the means of four replicates and expressed as oven-dried mass. Statistical analysis of the Cd concentrations was performed using the SPSS version 19.0. The comparison between the mean values of different treatments was carried out using two-way ANOVA, followed by Tukey’s test at a significance level of 5% (*p* < 0.05). The significance of linear regression was also defined by the SPSS as *p* < 0.05.

## 3. Results and Discussion

### 3.1. The Characters of the UGW-Biochar

#### 3.1.1. Structural Elements

The results from this experiment showed that the UGW-biochar was high in lignin. This was due to the UGW used in this study mostly consisting of tree trunks and branches from gardens. Moreover, our results showed that the structural element contents in the biochar derived from UGW were significantly influenced by the pyrolysis temperature (Table 2). For example, the biochar prepared at a high temperature (650 °C) showed greater O and N contents but a lower H content compared to the low temperature (350 °C), while the C content was not expected to change with temperature. The atomic ratio of H/C indicates the aromatization degree of the biochar, while the values of O/C and (O+N)/C indicate the functional groups containing O and N, respectively. The atomic ratio of H/C decreased with the increasing temperature, suggesting that a high pyrolysis temperature had a positive effect on the abundance of aromatic structures and a negative effect on hydroxyl abundance. The UGW650 had the highest degree of aromatics and might be more recalcitrant to decomposition compared to other biochars. Unlike the atomic ratio of H/C, the rise in pyrolysis temperature did not lead to the decrease in the atomic ratios of O/C and (O+N)/C. It was found that UGW450 and UGW650 had higher O/C and (O+N)/C values than UGW350 and UGW550. Bakshi et al. [33] proved that the pyrolysis process favors the elimination of H and O over C from the organic phase. Thus, increasing the pyrolysis temperature in our study drives the elimination of H and O toward completion. At the same time, increasing the pyrolysis temperatures also promotes the formation of inorganic ash material containing mostly carbonates, nitrates, and phosphates.

#### 3.1.2. Surface Analysis

**Figure 1:**
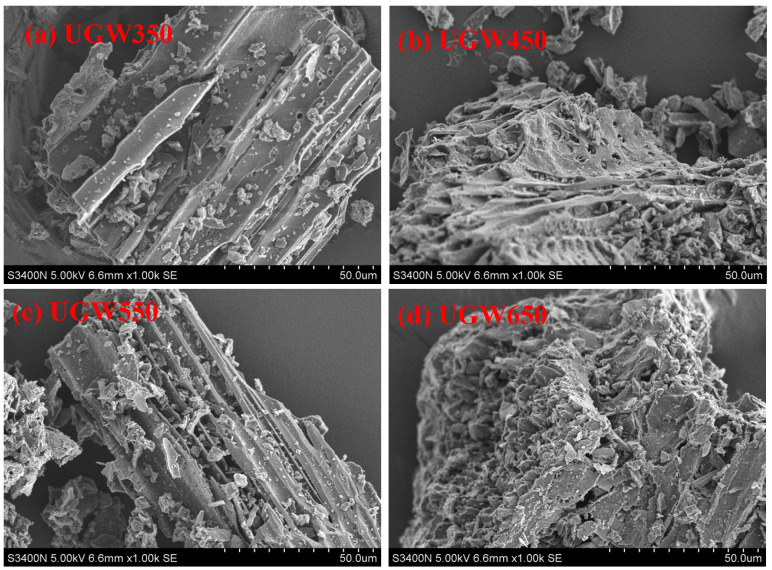
Scanning electron microscopy (SEM) images of urban greening waste-derived biochars. The (**a**) UGW350, (**b**) UGW450, (**c**) UGW550, and (**d**) UGW650 represent the biochars at pyrolysis temperatures of 350 °C, 450 °C, 550 °C, and 650 °C, respectively.

The results presented in Figure 1 show that all the prepared UGW-biochar had a porous structure. Moreover, they show that the surface morphology of higher-temperature biochars (Figure 1d) contain slit-shaped pores, whereas lower-temperature biochars (Figure 1a) show mostly plate-like particles with slit-shaped pores. The slit-shaped pores lead to a higher surface area.

Further, the results shown in Table 3 illustrate that the BET area, total pore volume, and micropore volume were improved with the increasing pyrolysis temperature (350 °C to 650 °C). A similar temperature effect on biochar has been reported in other studies [34,35,36]. For example, Zhang et al. [35] demonstrated the destruction of lignocellulosic structural components and well-developed micropores under higher temperatures; Zhu et al. [36] confirmed that the surface area was increased rapidly on biochars produced from 400 to 700 °C.

**Figure 2:**
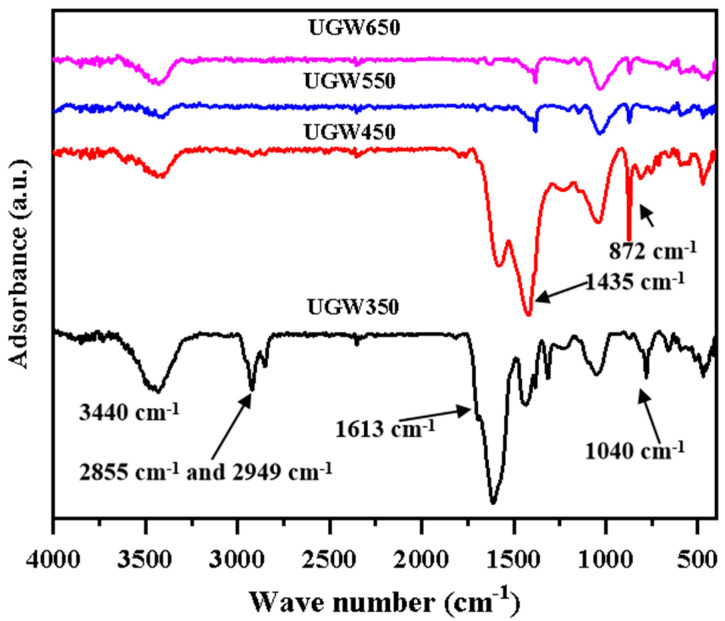
The Fourier transform infrared (FTIR) spectra of the biochar derived from UGW under different pyrolysis temperatures of 350 °C (UGW50), 450 °C (UGW450), 550 °C (UGW550), and 650 °C (UGW650), respectively.

The results presented in Figure 2 show that all the biochar prepared showed rich functional groups, such as O-H and N-H (3440 cm^−1^), aliphatic C-H (2855 cm^−1^), and C-H_2_ (2949 cm^−1^) in biopolymers [37], and carboxyl (1600 cm^−1^) and carbonyl (1700 cm^−1^) groups in esters bonds and aromatic rings [38]. However, biochar pyrolyzed above 450 °C showed the occurrence of two strong peaks at 1435 cm^−1^ and 872 cm^−1^, which confirmed the presence of an inorganic phase in the biochar derived from UGW [39]. Further, biochar formed at a high pyrolysis temperature (650 °C) led to the elimination of the polar groups, probably due to the thermal destruction of ester C=O and aromatic C=O, inducing the weak peaks of 1600 cm^−1^–1700 cm^−1^.

**Figure 3:**
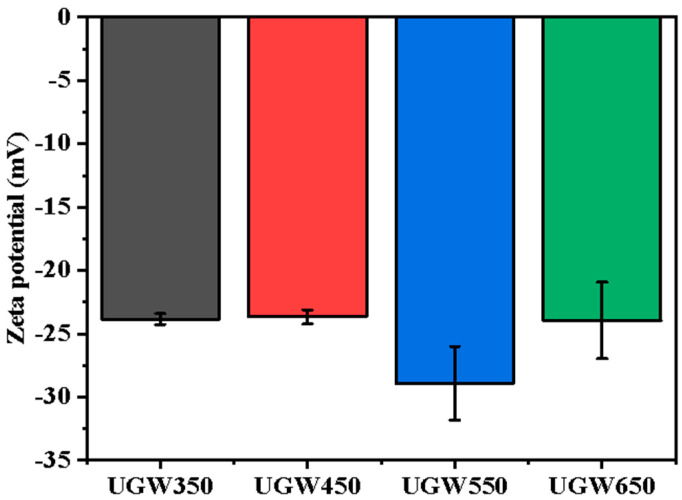
The zeta potentials of biochars derived from UGW under different pyrolysis temperatures of 350 °C (UGW50), 450 °C (UGW450), 550 °C (UGW550), and 650 °C (UGW650), respectively. Error bars are standard error of the means (*n* = 3).

The zeta potential of all biochars is shown in Figure 3. The results revealed that the zeta potential of biochar derived from UGW was influenced by the temperature, and ranged from −28.90 mV to −23.68 mV. The UGW450 has the highest zeta potential value. A previous study proved that the higher the zeta potential, the weaker the electronegativity of biochar [40]. A weaker surface electronegativity offers better crop protection since the low electronegativity results in aggregation and precipitation. Biochar can adsorb Cd and tends to precipitate due to agglomeration [41]. Thus, UGW450 was more suitable for the remediation of Cd-contaminated soils.

### 3.2. Adsorption Behavior of Cd Displayed by UGW Biochar

**Figure 4:**
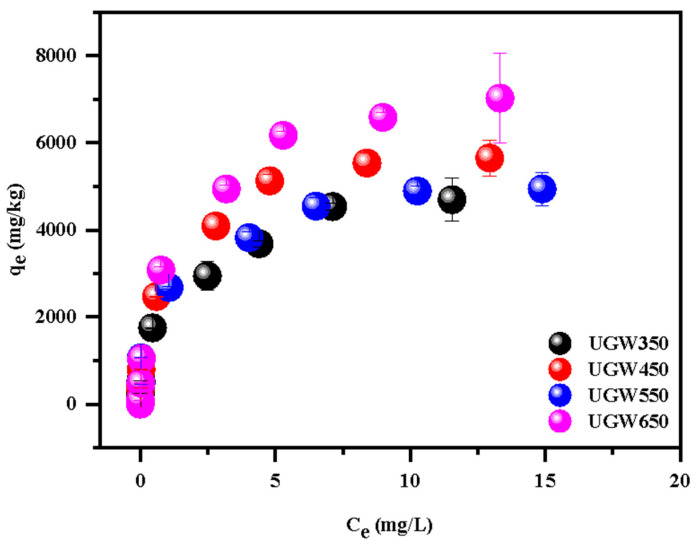
Adsorption isotherm of Cd onto biochar derived from UGW under different pyrolysis temperatures of 350 °C (UGW50), 450 °C (UGW450), 550 °C (UGW550), and 650 °C (UGW650), respectively. Error bars are standard error of the means (*n* = 3).

Figure S1 shows that the removal percentages of Cd by UGW-biochar were from 19.9% to 98.2% in the initial solution ranging from 0.06 mg/L to 19.00 mg/L. The isotherms of Cd on UGW-biochar are shown in Figure 4. The four UGW-biochars had similar behaviors in the isothermal adsorption of Cd and presented L-type isotherms with strong interactions even at low concentrations. The sorption isotherms of Cd on the biochars were simulated using the Langmuir, Freundlich, and Temkin equation (Table 4). The equations were used as follows:

(5) $$q_{e}=K_{F}C_{e}^{\frac{1}{n}}$$

(6) $$q_{e}=\frac{K_{L}q_{m}C_{e}}{1+K_{L}C_{e}}$$

(7) $$R_{L}=\frac{1}{\left(1+C_{0}K_{L}\right)}$$

(8) $$q_{e}=\frac{\mathrm{RT}}{b}\ln\left(K_{m}C_{e}\right)$$

where $C_{e}$ is the adsorption capacity, $q_{e}$ is the residual concentration of the adsorbate and $K_{F}$ and $K_{L}$ are the Freundlich and Langmuir adsorption coefficients, $\frac{1}{n}$ indicates the surface heterogeneity or adsorption intensity, $q_{m}$ represents the maximum adsorption capacity. $R_{L}$ is to determine whether the adsorption is favorable for Langmuir adsorption. $C_{0}$ is the lowest initial concentration of solutes in the solution, $K_{m}$ is the Temkin isotherm constant, b is the Temkin constant related to sorption heat, R is the universal gas constant, and T is the temperature in K.

In the literature, it is well established that the Freundlich model focuses on multilayer and heterogeneous adsorption [41]; the Langmuir on uniform adsorption without considering the interaction between the adsorbate and the adjacent site [34]; and the Temkin model, which presumes a multilayer adsorption process, considers interactions between the adsorbent and the adsorbate, but it ignores very small and very large concentration values [34]. As listed in Table 4, the slightly better fitting results were obtained from the Langmuir model, indicating that the adsorption mainly occurs on homogeneous surfaces.

The biochar prepared at higher pyrolysis temperatures tended to have larger values of q_m_, indicating a higher maximal adsorption capacity. The values of q_m_ followed the order UGW650 > UGW450 > UGW550 > UGW350. This is because UGW650 had the highest BET surface area and micropore volume. The better-developed pore structures at higher pyrolysis temperatures may have contributed to the biochar’s elevated adsorption capacity [42]. Meanwhile, the saturated adsorption capacity for UGW650, as suggested by q_m_, was 7431.96 mg/kg, comparable to some reported plant-derived biochars in the literature, i.e., 740 mg/kg–63,000 mg/kg [29]. This suggests that the UGW biochar would be applicable for the removal of Cd^2+^.

The zeta potential of biochar ranged from −28.90 mV to −23.68 mV. This indicated that biochar could adsorb positively charged Cd^2+^ due to the negative charge across its surface [43]. The FTIR spectrum in Figure 2 shows that biochar displays a variety of oxygen-containing surface groups (C=O, C-O, -OH), as well as others (olefins, -CH_2_, -CH_3_, aromatic rings). These oxygen-containing groups could form complexes with Cd and interact with these metals through electrostatic attraction and ion exchange [12].

However, the saturated adsorption capacity is commonly used as a proxy to estimate the potential of materials to remediate environmental pollution. This strategy has the potential to overestimate the materials’ capacity for remediation under the environmentally relevant Cd concentration (1.0–5.0 mg/L) [44]. Therefore, it is crucial to evaluate the adsorption capabilities under more pertinent environmental circumstances, i.e., a low-cadmium-content solution. It could be observed from Figure S1 that when the initial Cd concentration ranged from 0.06 to 0.83 mg/L, the removal percentage of the four UGW-biochars were similar. Moreover, Table 4 showed that the UGW450 tended to have larger values of K_L_, indicating a higher affinity for Cd, possibly due to the surface precipitation of compounds such as CdCO_3,_ Cd_3_P_2_, and Cd_3_(PO4)_2_. The FTIR spectrum of UGW450 showed a strong peak at 872 cm^−1^, which was attributed to the presence of carbonates. Table 1 further proved that UGW450 had the highest available P concentration and cation exchange capacity (CEC) of the UGW-biochars. Meanwhile, previous studies have indicated that biochars produced at low pyrolysis temperatures can effectively adsorb inorganic contaminants through the adsorption mechanisms of electrostatic interaction, precipitation, and ion exchange [45]. Biochars produced at low temperatures may improve the nutrient availability and crop yield in acidic and alkaline soils, whereas high-temperature biochar may enhance long-term soil carbon sequestration [46]. Combining the characterization and adsorption isotherms, it was concluded that UGW450 was considered to be more effective for the removal of Cd under relevant environmental Cd concentrations than UGW650, and it was selected as the material for Cd remediation in the pot experiments.

### 3.3. Conditioning Effect of UGW-Biochar on Cd-Contaminated Soils

#### 3.3.1. Soil Available Cd

Table 5 shows the effect of UGW-biochar on the three soils (Shaoguan, Yunfu, and Jiyuan) used in this experiment. The addition of the UGW450 treatment reduced the soil available Cd by 10.13%, 12.63%, and 18.81% in the Yunfu, Jiyuan, and Shaoguan soils, respectively, when compared to the control (Table 5). Table S1 showed that the biochar decreased the soil available Cd by 11.64–47.26%, depending on the dosage of the biochar. In our study, the high efficiency of the reduction in the available Cd concentration in the Shaoguan soil can be associated with the low pH value of 4.8, as delineated in Table 5. In acidic soil environments, Cd is predominantly present in the soil solution as inorganic cationic species, such as free Cd (II). An increase in pH has induced a substantial transformation of Cd into organic and inorganic neutral species [47]. In this case, applying UGW450 elevated the pH in the Shaoguan soil from 4.8 to 5.9. This suggests that UGW450 effectively facilitated the conversion of acid-soluble and reducible Cd into more stable residual forms, thus diminishing its availability and mobility within the soil matrix. Furthermore, Cd has the potential to form precipitates with phosphate and other anions on biochar surfaces, with the solubility of these compounds decreasing concomitantly as the pH rises. Consequently, incorporating UGW450 into the soil matrix mitigated the concentration of bioavailable Cd in acidic soil conditions.

In terms of the Jiyuan and Yunfu soil types, which were classified as neutral soils, the performance of the biochar was mainly influenced by the Cd concentration in the different soils. The total Cd content in Jiyuan soils was higher than in the Yunfu soil (Table 1). According to Meili Xu’s classification criteria [23], the Yunfu soil was categorized as lightly Cd-contaminated soil, while the soil in Jiyuan was classified as heavily Cd-contaminated soil. When assessing the reduction efficiency in these two soil types, UGW450 exhibited a markedly more significant reduction in the bioavailable Cd in the Jiyuan soil relative to the Yunfu soil. This observation is in agreement with the findings by Xu et al. [23], who reported that the addition of biochar to agricultural soils demonstrated higher efficiency in medium and heavily Cd-contaminated soil than in lightly Cd-contaminated soils. In medium and heavily Cd-contaminated soil, biochar primarily reduced Cd availability through its direct effect. Consequently, the direct adsorption of Cd by biochars resulted in a more pronounced reduction in medium and heavily Cd-contaminated soils as opposed to lightly Cd-contaminated soil [48].

#### 3.3.2. Cabbage Biomass and Cd Accumulation

**Figure 5:**
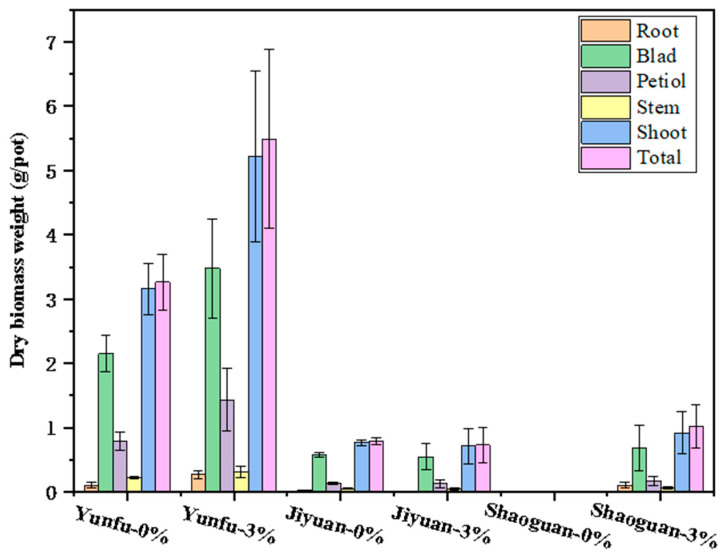
The dry biomass weight of cabbage grown in the soils treated with 0% and 3% UGW450 (in mass) and then grown with cabbage. Error bars are standard error of the means (*n* = 3).

Figure 5 shows the dry matter of cabbages cultivated in soils with 0% and 3% biochar treatments. In the Shaoguan (acidic) soil, the application of the UGW450 treatment from Foshan City increased the total dry biomass from 0 g/pot (plants died) to 1.02 g/pot, with the shoot and root dry masses reaching 0.92 g/pot and 0.10 g/pot, respectively. It seems that the UGW450 treatment effectively reduced the toxicity of Cd in the Shaoguan soil, which might affect cabbage growth. Previous studies have shown that the application of biochar can bolster Cd adsorption and diminish the quantities of soluble and leachable Cd in the soil, in turn reducing the concentration of Cd accessible for uptake by cabbage roots and shoots, subsequently promoting plant growth and cellular division [12]. This mechanism is evidenced by the reduction in the available Cd concentration in the soil from 3.03 mg/kg to 2.46 mg/kg in this study.

The other two soils (Jiyuan and Yunfu) were classified as neutral soils. The results presented in Figure 5 show that the application of UGW450 induced differences in terms of biomass yield. In the case of the Jiyuan soil, the UGW450 treatment decreased the shoot and root dry biomass of the cabbage by 6.6% and 50.0%, respectively, relative to the control. This resulted in an overall reduction of 7.6% in the total biomass weight compared to the control. This reduction is likely attributed to the high concentration of Cd in Jiyuan soils, which could have exerted a toxic effect on the cabbage growth.

In terms of the Cd concentration samples from the Jiyuan soil, the application of UGW450 significantly increased the Cd concentration in the blade and stem by 22.9% and 1.3%, respectively, relative to the control (Table 6). This resulted in an overall increase of 19.6% in the Cd concentration in the shoot compared to the control (Table 6). The lack of effectiveness in reducing the Cd uptake with this treatment can be demonstrated, even though biochar can reduce the Cd bioavailability while the remaining Cd concentrations in the soil remain toxic. Shen et al. [24] found that the Cd content significantly (*p* < 0.05) increased in the leaves (109.3–93.6%), stem (68.8–47.9%), and root (75.8–41.4%) with the addition of biochar. This enhanced Cd content of the plants was probably attributed to the dissolved organic matter (DOM) released from biochar. The application of biochar released large amounts of DOM into soils, and the DOM bonded with metals to form DOM–metal complexes, which increased the mobility of the metals in soils and the uptake of the metals by plants [49,50]. Xu et al. [23] reported that biochar directly interacted with Cd in medium (4.18 mg/kg) and heavily Cd-contaminated soils (10.01 mg/kg), whereas it indirectly interacted with Cd in lightly Cd-contaminated soil (0.46 mg/kg). As listed in Table 1, the total Cd of the Jiyuan soil was 14.02 mg/kg, which belonged to the group of heavily Cd-contaminated soils. It is extremely possible that the DOM from UGW450 directly bonded with Cd to form DOM–Cd complexes, which increased the content of Cd in the shoot.

The application of the UGW450 treatment in the Yunfu soil increased the shoot and root dry biomass by 65.2% and 156.8%, respectively, compared to no-biochar soils, while the total biomass weight increased by 68.1% relative to the no-biochar soil. The increase in biomass weight was mainly due to an increase in the blade and petiole (Figure 5). Additionally, UGW450 led to a decrease in the concentration of Cd in cabbage. The concentrations of Cd in the root and shoot decreased by 42.7% and 15.4%, respectively, after the UGW450 treatment, as listed in Table 6. There was a slight decrease in the content of Cd in the cabbage blade after the addition of 3% biochar. The concentrations of Cd in the blade decreased by 13.0%. The overall concentration of Cd in the total biomass was slightly decreased by 17.3%.

**Figure 6:**
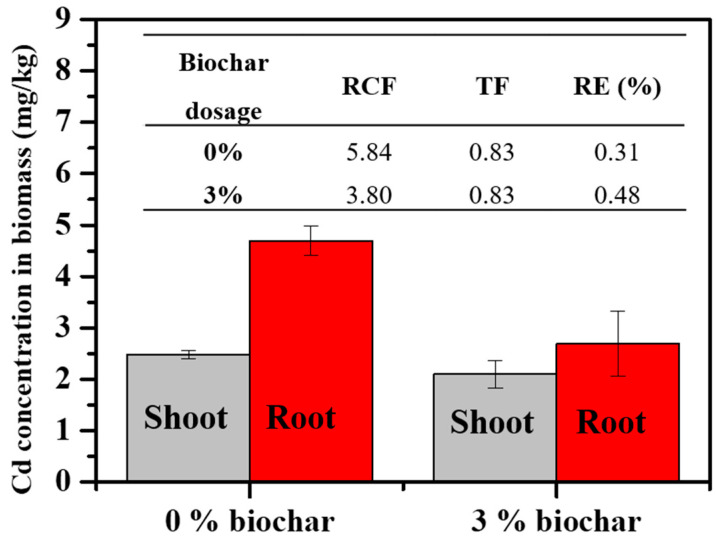
The concentration of Cd in cabbage biomass; the bioaccumulation factor (BCF), translocation factor (TF), and removal efficiency (RE) were calculated from the concentration of Cd in the biomass grown in Yunfu soil. Error bars are standard errors of the means (*n* = 3).

Given that the cabbage in Yunfu soils resumed normal growth after the UGW450 treatment, we further examined the concentration and translocation of Cd within the Yunfu soil–plant system. The bioaccumulation factor (BCF), translocation factor (TF), and removal efficiency (RE) were calculated and are shown in Figure 6. It is evident that the concentration of Cd in the roots significantly decreased after the addition of biochar to the soil. Furthermore, the BCF dropped from 5.84 to 3.80 as the biochar dosage increased from 0% to 3%, indicating that the UGW-biochar amendment impacted the bioaccumulation of soil Cd in cabbage roots. Although the TF value remained unchanged, the reduction of Cd in the roots also decreased the Cd content in shoots. Consequently, the concentration of Cd in the shoots diminished from 2.48 mg/kg to 2.10 mg/kg. Additionally, the RE experienced a slight increase, primarily attributable to the relatively high increase in biomass.

Based on the results presented, it can be concluded that the application of UGW450 biochar exhibited varying effects on soils with distinct pH values and pollution levels. In the acidic soil, the addition of UGW450 increased cabbage biomass. In contrast, in the two types of neutral soil, the biomass assay revealed conflicting results. In lightly Cd-contaminated soil, the addition of UGW450 significantly increased the cabbage biomass and reduced the Cd content in both soil and plants while also influencing the bioaccumulation of Cd in the soil–plant system. Conversely, in the heavily Cd-contaminated soil, the inclusion of UGW450 did not substantially enhance the cabbage biomass or alleviate soil Cd toxicity. As a result, the efficacy of the UGW450 application is contingent upon soil pH and the extent of Cd contamination.

In summary, the UGW450 biochar positively impacted the growth of cabbage in Cd-contaminated soil and could reduce the Cd content in both the soil and plants. However, this effect was dependent on the soil pH and Cd concentration in the soil. These findings suggest that biochar possesses potential value in the remediation of Cd-contaminated soil but requires adjustment and optimization according to specific circumstances.

## 4. Conclusions

In this investigation, UGW was utilized to generate UGW-biochar for the remediation of Cd-contaminated agricultural soils. By analyzing the properties of UGW-biochar at various temperatures, it was found that UGW biochar, specifically when pyrolyzed at 450 °C (termed UGW450), demonstrated the highest CEC values, zeta potential values, and an enhanced binding affinity towards Cd, particularly at low Cd equilibrium concentrations. Consequently, UGW450 was selected as the preferred material for Cd remediation within a Chinese cabbage soil ecosystem. In acidic Shaoguan soil, the deployment of UGW450 markedly increased cabbage biomass. Conversely, in neutral soils such as Jiyuan and Yunfu, there was a noticeable decrease in biomass growth. Additionally, our findings indicate that in soil contaminated with low levels of Cd (0.79 mg/kg), UGW450 significantly elevated the cabbage biomass and concurrently reduced Cd concentrations in both the soil and plant. However, under medium-level Cd contamination (5.86 mg/kg), UGW450′s effectiveness in enhancing the cabbage biomass and mitigating soil Cd toxicity was limited, evidenced by a minor increase in plant Cd levels. In summary, UGW450 biochar, as utilized in this study, demonstrates promising potential for the remediation of Cd-contaminated soils. However, its application should be tailored and optimized for specific environmental conditions.

## Figures and Tables

**Table 1:** Selected properties of the soils and biochar used.

Country	Yunfu	Jiyuan	Shaoguan	-	-	-	-
Soil type	Udept	Ustalf	Udult	-	-	-	-
Biochar	-	-	-	UGW350	UGW450	UGW550	UGW650
Total N (g/kg)	3.41	2.01	1.73	13.03	12.31	10.26	9.24
Total P (g/kg)	1.24	1.39	1.25	2.90	3.61	3.74	4.33
pH(H_2_O)	7.5	7.4	4.8	7.92	9.77	9.89	10.15
Total Cd (mg/kg)	1.97	14.02	4.16	0.35	0.25	0.22	0.25
Available Cd (mg/kg)	0.79	5.86	3.03	-	-	-	-
Available P (mg/kg)	-	-	-	560.6	784.8	271.3	220.9
CEC (cmol/kg)	-	-	-	6.7	10.7	3.9	4.4

**Table 2:** Structural elements in the biochar prepared from urban greening waste under different pyrolysis temperatures of 350 °C (UGB350), 450 °C (UGB450), 550 °C (UGB550), and 650 °C (UGB650), respectively.

Biochar	C%	H%	O%	N%	H/C	O/C	(O+N)/C
UGW350	58.24	4.09	35.85	0.15	0.84	0.46	0.64
UGW450	56.27	2.95	39.17	0.21	0.63	0.52	0.72
UGW550	59.32	2.44	36.68	0.22	0.49	0.46	0.64
UGW650	55.08	1.96	41.84	0.24	0.43	0.57	0.78

**Table 3:** The porosity of the biochar derived from UGW under different pyrolysis temperatures of 350 °C (UGW50), 450 °C (UGW450), 550 °C (UGW550), and 650 °C (UGW650), respectively.

Biochar	BET Surface Area	Total Pore Volume at P/P_0_ = 0.985	Micropore Volume	Average Pore Diameter
	m^2^/g	cm^3^/g		nm
GB350	1.7	4.7 × 10^−3^	2.6 × 10^−4^	11.1
GB450	2.2	9.2 × 10^−3^	4.6 × 10^−4^	16.3
GB550	2.2	18.9 × 10^−3^	6.0 × 10^−4^	34.7
GB650	3.5	13.4 × 10^−3^	7.1 × 10^−4^	15.5

**Table 4:** The model fitting parameters of Cd isothermal adsorption by the biochars derived from UGW under different pyrolysis temperatures of 350 °C (UGW50), 450 °C (UGW450), 550 °C (UGW550), and 650 °C (UGW650), respectively.

Model	Parameters	UGW350	UGW450	UGW550	UGW650
Freundlich Isotherm	K_F_ (L/mg)	2177.41	2875.97	2516.99	3287.77
	1/n	0.34	0.30	0.28	0.32
	R^2^	0.96	0.97	0.95	0.98
Langmuir Isotherm	q_m_ (mg/kg)	5030.72	6011.15	5198.63	7431.96
	K_L_ (L/mg)	0.81	1.07	1.00	0.85
	R_L_	0.87	0.98	0.96	0.97
	R^2^	0.97	0.98	0.96	0.98
Temkin Isotherm	b (J/mol)	4.69	3.81	4.40	2.98
	K_m_ (L/g)	247.72	296.53	303.13	184.21
	R^2^	0.92	0.94	0.95	0.94

**Table 5:** The properties of the soils that were treated with 0% and 3% UGW450 (in mass) and then grown with cabbage.

Soils	Biochar Addition (%)	Soil pH	Available Cd (mg/kg)
Yunfu	0	7.5 ± 0.3	0.79 ± 0.17
Yunfu	3	7.5 ± 0.5	0.71 ± 0.15
Jiyuan	0	7.4 ± 0.3	5.86 ± 0.26
Jiyuan	3	7.5 ± 0.2	5.12 ± 0.22
Shaoguan	0	4.8 ± 0.1	3.03 ± 0.32
Shaoguan	3	5.9 ± 0.2	2.46 ± 0.19

**Table 6:** The concentrations of Cd in the blade, petiole, stem, shoot, root, and total biomass of cabbage with 0% and 3% UGW450 (in mass).

Soils	Biochar Addition (%)	Blade (Cd) (mg/kg)	Petiol (Cd) (mg/kg)	Stem (Cd) (mg/kg)	Shoot (Cd) (mg/kg)	Root (Cd) (mg/kg)	(Total Cd) (mg/kg)
Yunfu	0	2.93 ± 0.07 gh	1.58 ± 0.32 gh	1.31 ± 0.20 h	2.48 ± 0.08 gh	4.69 ± 2.80 fgh	2.57 ± 0.14 gh
	3	2.55 ± 0.36 gh	1.18 ± 0.16 h	1.24 ± 0.25 h	2.10 ± 0.27 gh	2.69 ± 0.63 gh	2.13 ± 0.24 gh
Jiyuan	0	65.63 ± 14.90 bc	29.58 ± 2.96 d	22.01 ± 3.25 de	56.57 ± 11.80 c	NA	NA
	3	80.65 ± 23.56 a	25.18 ± 9.11 d	22.30 ± 4.14 de	67.67 ± 19.86 b	NA	NA
Shaoguan	0	NA	NA	NA	NA	NA	NA
	3	13.35 ± 2.49 ef	6.78 ± 1.13 fgh	5.37 ± 1.70 fgh	11.24 ± 2.33 fg	NA	NA

NA: the concentration of Cd could not be determined due to the low biomass. Each value represents the mean ± standard error of the means. Two-way variance analysis (two-way ANOVA) was carried out and followed by the Tukey HSD test. The different letters (a–h) showed significant differences between experiment treatments at *p* < 0.05, *n* = 3.

## Data Availability

The data presented in this study are available on request from the corresponding author. The data are not publicly available due to privacy.

## Supplementary Materials

The following supporting information can be downloaded at https://www.mdpi.com/article/10.3390/toxics12010008/s1, Figure S1: The removal percentage of Cd by UGW-biochar; Table S1: Literature comparison for biochar selection; Table S2: The comparison of WHO/FAO Cd permissible limits values with our soil-plant system values.
