# Supplementary material for: Biochar Derived from Urban Green Waste Can Enhance the Removal of Cd from Water and Reduce Soil Cd Bioavailability
Source: Toxics. 2023 Dec 21;12(1):8. doi: 10.3390/toxics12010008 (PMC10819508; doi:10.3390/toxics12010008)
Supplement: Supplementary file 1 [file toxics-12-00008-s001.zip › toxics-2757617-supplementary.pdf]

Supporting information

Biochar Derived from Urban Green Waste Can Enhance the Removal of Cd from Water and Reduce Soil Cd Bioavailability

Xiang Li [1], Paramsothy Jeyakumar [2], Nanthi Bolan [3,4], Lianxi Huang [1], Muhammad Saqib Rashid [1], Zhongzhen Liu [1,*], Lan Wei [1,*] and Hailong Wang [5,6]

1 Key Laboratory of Plant Nutrition, and Fertilizer in South Region, Ministry of Agriculture, Guangdong Key Laboratory of Nutrient Cycling and Farmland Conservation, Institute of Agricultural Resources and Environment, Guangdong Academy of Agricultural Sciences, Guangzhou 510640, China; lixiang142213@163.com (X.L.); hlx4@163.com (L.H.); saqibssr@mail.ustc.edu.cn (M.S.R.)
2 Environmental Sciences, School of Agriculture & Environment, Massey University, Private Bag 11 222, Palmerston North 4442, New Zealand; p.jeyakumar@massey.ac.nz
3 UWA School of Agriculture and Environment, The University of Western Australia, Perth, WA 6009, Australia; nanthi.bolan@uwa.edu.au
4 The UWA Institute of Agriculture, The University of Western Australia, Perth, WA 6009, Australia
5 School of Environmental and Chemical Engineering, Foshan University, Foshan 528000, China; hailong.wang@fosu.edu.cn
6 Guangdong Provincial Key Laboratory of Integrated Agro-Environmental Pollution Control and Management, Institute of Eco-Environmental and Soil Sciences, Guangdong Academy of Sciences, Guangzhou 510650, China
* Correspondence: lzzgz2001@163.com (Z.L.); weilan@gdaas.cn (L.W.)

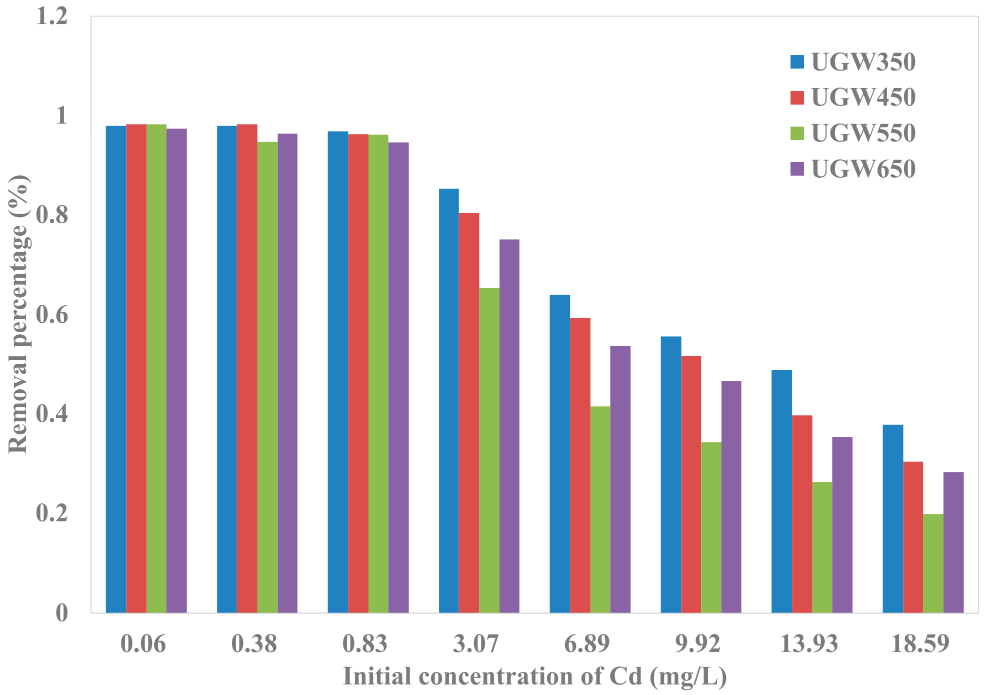

Figure S1 The removal percentage of Cd by UGW-biochar.

Table S1. Literature comparison for biochar selection.

Biochar %	Contaminants	Crop/ soil	Reduction of soil available Cd	References
2.5%-5%	Cd	rice	30.85–47.26%	Biochar-mediated Cd accumulation in rice grains through altering chemical forms, subcellular distribution, and physiological characteristics(DOI: 10.1007/s42773-023-00248-4)
1%-15%	Cd	maize	11.64%-16.3%	Effects of biochars derived from chicken manure and rape straw on

speciation and phytoavailability of Cd to maize in artificially contaminated loess soil(DOI: 10.1016/j.jenvman.2016.10.020)

Table S2 The comparison of WHO/FAO Cd permissible limits values with our soil-plant system values.

Heavy metal	Soil (mg/kg) permissible limit WHO/FAO	Plant (mg/kg) limit WHO/FAO	Reference	This study (mg/kg)
Cd	3.00	0.21	• DOI:<br>• 10.1007/978-1-4020-9139-1_2<br>• 10.1080/23311843.2017.1405887	❖ Soil:<br>1) Yunfu: 0.71<br>2) Jiyuan: 5.12<br>3) Shaoguan: 2.46<br><br>❖ Plants > 0.21 (Not edible)
